# ProAlgaZyme subfraction improves the lipoprotein profile of hypercholesterolemic hamsters, while inhibiting production of betaine, carnitine, and choline metabolites

**DOI:** 10.1186/1743-7075-10-55

**Published:** 2013-08-27

**Authors:** Andreea Geamanu, Arvind Goja, Nadia Saadat, Pramod Khosla, Smiti V Gupta

**Affiliations:** 1 Nutrition and Food Science, 3009 Science Hall, Wayne State University, Detroit, MI 48202, USA

**Keywords:** Apolipoprotein A1, Betaine, Carnitine, Choline, HDL, ^1^H NMR, Lipid metabolism, Metabolomics, ProAlgaZyme

## Abstract

**Background:**

Previously, we reported that ProAlgaZyme (PAZ) and its biologically active fraction improved plasma lipids in hypercholesterolemic hamsters, by significantly increasing the high density lipoprotein cholesterol (HDL-C) while reducing non-HDL cholesterol and the ratio of total cholesterol/HDL-C. Moreover, hepatic mRNA expression of genes involved in HDL/reverse cholesterol transport were significantly increased, while cholesteryl ester transfer protein (CETP) expression was partially inhibited. In the current study, we investigated the therapeutic efficacy of the biologically active fraction of PAZ (BaP) on the plasma lipid and plasma metabolomic profiles in diet induced hypercholesterolemic hamsters.

**Methods:**

Fifty male Golden Syrian hamsters were fed a high fat diet for 4 weeks prior to randomization into 6 groups, based on the number of days they received subsequent treatment. Thus animals in T0, T3, T7, T10, T14, and T21 groups received BaP for 0, 3, 7, 10, 14, and 21 days, respectively, as their drinking fluid. Plasma lipids were assayed enzymatically, while real-time reverse transcriptase polymerase chain reaction (RT-PCR) provided the transcription levels of the Apolipoprotein (Apo) A1 gene. The plasma metabolomic profile was determined using ^1^H nuclear magnetic resonance (NMR) spectroscopy in conjunction with multivariate analysis.

**Results:**

Plasma HDL-C was significantly increased in T3 (P < 0.05) and T21 (P < 0.001), while non-HDL cholesterol was significantly reduced in T3, T7, T10 (P < 0.001) and T14, T21 (P < 0.01). Moreover, the ratio of total cholesterol/HDL-C was significantly lower in all BaP treated groups (P < 0.001) as compared with T0. Quantitative RT-PCR showed an increase in Apo A1 expression in T10 (3-fold) and T21 (6-fold) groups. NMR data followed by multivariate analysis showed a clear separation between T0 and T21 groups, indicating a difference in their metabolomic profiles. Plasma concentrations of metabolites associated with a risk for atherosclerosis and cardiovascular disease, including choline, phosphocholine, glycerol-phosphocholine, betaine and carnitine metabolites were significantly lower in the T21 group.

**Conclusion:**

Treatment with BaP significantly improved the plasma lipid profile by increasing HDL-C and lowering non-HDL cholesterol. In addition, BaP potentially improved the plasma metabolomic profile by reducing the concentration of key metabolites associated with risk for atherosclerosis and cardiovascular disease.

## Introduction

The etiology of cardiovascular disease (CVD) has been strongly linked with elevated levels of plasma low density lipoprotein cholesterol (LDL-C), accompanied by reduced high density lipoprotein cholesterol (HDL-C) concentrations [1]. Despite considerable advancements in therapeutics to improve the plasma lipoprotein profile, from statin drugs to dietary modifications, it is hard to achieve a decline in cardiac events in practice, due to the difficulties associated with determining optimal targets for such interventions. More recently, the interest in functional foods (including some seaweed extracts, freshwater and marine algal products), has drawn the attention of the scientific community, due to their ability to decrease plasma cholesterol and induce genetic modifications with respect to lipoprotein metabolism [2-5]. ProAlgaZyme (PAZ) is the fermentation product of different freshwater organisms, mainly green and red algae, and consists of a mixture of approximately 90% salts and 10% organic components [6]. The therapeutic effect of PAZ on body weight, body mass index, fasting blood glucose, blood lipids, and markers of inflammation was first investigated by Oben et al. in patients with metabolic syndrome [6]. These investigators concluded that PAZ could be used as a dietary agent for prevention of CVD, as the results showed a positive effect on various parameters analyzed [6]. To better understand the effect of PAZ on lipid metabolism, and to elucidate the mechanism by which the levels of HDL-C were increased, we previously reported a study that analyzed different subfractions of PAZ for their potential preventative effect on lipids, when administered simultaneously to animals on a high fat diet. Hepatic mRNA expression was also analyzed to determine the effect of PAZ on genes involved in HDL/reverse cholesterol transport in diet induced hypercholesterolemic hamsters [7]. The goal of the current study was to determine if the plasma lipid profile in established hypercholesterolemic animals could be improved by therapeutic intervention with the biologically (BaP) active fraction documented in the previous study. Secondly, the current study aimed to determine the time course required for the effect on lipid parameters.

However, as observed in recent reports, the lipid profile does not provide a complete picture of disease progression. Analysis of a multifactorial disease, such as atherosclerosis and other forms of CVD, using a limited number of biomarkers can lead to inaccurate diagnosis and treatment regimes, weakening the advancement of new therapies. In contrary, the omics-based approaches (metabolomics, in particular) have allowed scientists to characterize, at the molecular level, complex biological systems and their changes in pathological processes [8,9], providing an excellent tool for examining phenotypes using descriptors for hundreds of metabolites. Metabolomics analysis aims to detect changes in the relative concentrations of endogenous small molecules that characterize the changes in metabolism, thus helping to reveal the metabolic state of biological systems [10]. The metabolomic strategy has been successfully used to investigate multiple distinct biological processes including toxicological effects [11,12], disease processes [13], and nutritional interventions [14,15]. Recently, nutritional metabolomics has provided important insights into understanding metabolic responses related to diet-induced hypercholesterolemia and atherosclerosis [10,16].

Therefore, in addition to studying the effect of BaP on the lipid profile, we used ^1^H nuclear magnetic resonance (NMR) spectroscopy, a powerful tool for simultaneous analysis of several hundreds or thousands of metabolites in biological fluids such as urine or plasma [17], to get a deeper insight into the metabolomic shift over time. Thus, lipid profiles were correlated with the changes in plasma metabolomic profiles. Data were analyzed using multivariate analysis SIMCA P + software and targeted profiling was applied to indentify and quantify the metabolites that were different between the treatment and control groups. Here we report the changes in the lipid profile as compared and correlated with perturbations in the plasma metabolomic profile due to dietary intake of BaP.

## Materials and methods

### Animals and diets

Fifty 8-week-old male Golden Syrian hamsters (*Mesocricetus auratus*), LVG strain (Charles River Laboratories, Wilmington, MA), each weighing approximately 80 g, were randomized into six groups: T0 (n = 10) and T3, T7, T10, T14, and T21 (n = 8 per group). Upon arrival, they were acclimatized and given water and laboratory rodent diet 5001 (Lab Diet, Richmond, IN) *ad libitum* for one week prior to the initiation of the experimental study. For the subsequent four weeks, hamsters were fed a high fat diet containing 30% calories from fat (Dyets Inc., Bethlehem, PA, Table 1). Distilled water was provided as their drinking fluid. The composition of the high fat diet was shown to induce a hypercholesterolemic state in the hamsters in our previous study [7]. After four weeks, the hypercholesterolemic hamsters were provided with a 20% (v/v) solution of BaP, as their drinking fluid [7]. The process of fractionation of the PAZ has been detailed previously [7]. BaP was administrated for 0 days (T0 group), 3 days (T3 group), 7 days (T7 group), 10 days (T10 group), 14 days (T14 group), and 21 days (T21 group), respectively. Body weight as well as food and fluid intake were recorded weekly. Animals were housed individually in a temperature-controlled room (25°C) maintained on a 12-h light/dark cycle. The study protocol was approved by the Institutional Animal and Care Use Committee at Wayne State University, Detroit.

**Table 1:** Composition of the experimental custom purified diet with coconut oil and soybean oil

**Ingredient**	**kcal./gm**	**grams/kg**
Casein	3.58	110
Lactalbumin	3.9	110
L-Arginine	4	2.5
L-Tryptophan	4	0.3
Cornstarch	3.6	370.2
Dyetrose	3.8	175
Coconut Oil	9	138.6
Soybean Oil	9	1.4
TBHQ	0	0.028
Cellulose	0	44
Cholesterol	0	1
Mineral Mix #260001*	0	35
Vitamin Mix #360001**	3.84	10
Choline Bitartrate	0	2

Abbreviation: *TBHQ* Tertiary Butylhydroquinone.

* **Hamster Mineral mix** prepared by Dyets, Inc.

** **Hamster Vitamin Mix** prepared by Dyets, Inc.

### Plasma and tissue collection

Each group of hamsters was sacrificed at different time points, based on the number of days of treatment with BaP. Hamsters were fasted for 8 hours and anesthetized under CO_2_/O_2_ (50:50) gas (Metro Welding, Detroit, MI) prior to sacrifice. Blood was collected on ice by cardiac puncture with syringes previously rinsed with potassium EDTA solution (15% wt/v) and plasma was immediately separated after centrifugation at 1000 × g for 15 minutes at 4°C. Liver tissues were collected and immediately flash frozen in liquid nitrogen for further analyses.

### Plasma lipid analysis

Plasma triglycerides (TG) and total cholesterol (TC) concentrations were determined enzymatically, while HDL-C was measured in the supernatant following precipitation with the Mg ^2+^/dextran sulfate according to the manufacturer’s protocol (Pointe Scientific, Canton, MI), as detailed previously [7]. The concentration of non-HDL cholesterol was calculated as the difference between the measured TC and HDL-C, and includes the sum of very low density lipoprotein, intermediate density lipoprotein, and LDL-C.

### Real-time reverse transcription polymerase chain reaction (RT-PCR)

Total RNA from the liver was extracted using miRNeasy Mini Kit (Qiagen, Valencia, CA) and reverse transcription of RNA into cDNA was performed using the High Capacity mRNA to cDNA Master Mix kit (Applied Biosystems, Carlsbad, CA) as per the manufacturer’s protocol. Total RNA was obtained by adding 40 μL RNAase-free H_2_O followed by centrifugation at 1000 × g for 1 minute. mRNA was further subjected to reverse transcription using the High-Capacity RNA to cDNA Master Mix kit. The reaction was carried in a total of 20 μL mixture (4 μL of Complete Master Mix, 8 μL of total RNA, and 8 μL of nuclease-free H_2_O) in a Master cycler (Eppendorf, Hauppauge, NY). The program was set for 5 minutes at 25°C, 30 minutes at 42°C, 5 minutes at 85°C, and 1 hour at 4°C. A 2 μL sample of the obtained cDNA was used for each real-time RT-PCR reaction using SYBR Green Master Mix (Applied Biosystems) and an MX3005P instrument (Strategene, Santa Clara, CA) to determine the relative transcription levels of Apo A1(F: 5′-ACC-GTT-CAG-GAT-GAA-AAC-TGT-AG-3′, R: 5′-GTG-ACT-CAG-GAG-TTC-TGG-GAT-AAC-3′) [18]. The cycle conditions were 10 min at 95°C followed by 40 cycles of incubation at 95°C for 15 seconds, then 60°C for 1 minute. No accumulations of nonspecific products or primer dimers were observed using nontemplate control wells, as a result of prior optimization with the concentration used. The data were analyzed according to the comparative threshold cycle (C_t_) method and normalized by glyceraldehydes-3-phosphate dehydrogenase (GAPDH) expression in each sample. Levels of mRNA expression were reported as fold differences compared with hamsters fed the high fat diet and water.

### Plasma metabolomic analysis using ^1^H NMR

Plasma samples were diluted with deuterium oxide (D_2_O) in a 4:1 ratio, and a reference buffer solution (NMR solvent) containing 5 mmol/L disodium-2,2-dimethyl 2-silapentane-5-sulphonate and 10 mmol/L imidazole in D_2_O (Sigma-Aldrish, Mississauga, ON) was added in a 9:1 ratio (9 parts of diluted plasma sample : 1 part NMR solvent). After preparation, samples were transferred to 5 mm NMR tubes (Sigma-Aldrich, St. Louis, MO) and ^1^H NMR spectra were acquired on a 600 MHz Agilent spectrometer, operating at 599.773 MHz frequency and a temperature of 300 K [10,19,20]. One-dimensional NMR spectra were acquired using the Carr-Purcell-Meiboom-Gill (CPMG) spin-echo sequence with presaturation, to attenuate broad signals from proteins and lipoproteins. Application of the CPMG pulse sequence resulted in spectra with signals from the small molecular weight metabolites only, due to their longer transverse relaxation time. These spectra were measured using a spin-echo loop time of 0.16 s and a recycling time of 14 s. A total of 64 scans were collected using a spectral width of 10 ppm and an acquisition time of 4 s. The NMR data were processed using ACD/Spec Manager 7.00 software (Advanced Chemistry Development Inc., Toronto, Canada) and free induction decay files were obtained. The acquired NMR spectra were processed by editing, auto-phasing, and auto-baseline correction. Intelligent binning was used to divide the edited spectra into 1000 bins. The spectra were digitized to a table of common integrals and exported as a non-negative value text file for multivariate data analysis.

### Multivariate data analysis

Data were statistically analyzed by principal component analysis (PCA), partial least square (PLS) and PLS-discriminant analysis (PLS-DA), using SIMCA-P + 13.0 software (Umetrics, Umea, Sweden). PCA is an unsupervised multivariate projection method designed to extract and display the systemic variation in the data matrix X as a score plot [21]. The corresponding loading plot provides information about the part of the spectrum that is responsible for the similarities and/or dissimilarities in the data set as observed in the score plot. Moreover, the orthogonal projections to latent structures (OPLS) aids in the process of identifying statistically significant and potentially biochemically significant metabolites based on contribution to the model and their reliability. PLS is a regression extension of PCA, which is used to connect the information in two blocks of variables, X and Y, to each other [19]. In PLS-DA the data set is distributed into classes and its objective is to find a model that separates the classes of observation on the basis of their X-variables, while using a hypothetical Y-variable. Both PLS and PLS-DA methods of analysis are supervised, which implies that some information about the data set is provided to the software prior to analysis [21]. PLS was used to correlate the results from the ^1^H spectra with other measured factors (TG, TC, HDL-C, TC/HDL-C, non-HDL cholesterol), keeping these factors as Y parameters. All data were Pareto-scaled prior to analysis.

Each point on the score plots corresponds to an individual animal, to expose specific grouping and the relationship between the samples. Each dot on the loading plot represents a single NMR spectral area or bin, illustrating the significance of each metabolite for the variation described [22].

Identification and quantification of the metabolites in spectra was accomplished using Chenomx NMR Suite 7.6 software (Chenomx, Alberta, Canada) [23], focusing on the peaks of the spectra that differentiate the groups. Chenomx software uses targeted profiling to reduce analysis time, combining advanced analysis tools with a compound library of approximately 300 metabolites [24]. Once the changes in metabolite concentrations had been determined, the pathways affected by the respective metabolites were identified using online KEGG database.

### Statistical analysis

All data evaluated the differences between the control and treatment groups and were expressed as mean ± standard error. The data were analyzed to determine the effect of BaP relative to distilled water, while the animals were fed a high fat diet. The significance of anthropometric measurements and plasma lipid concentrations were determined using one-way ANOVA tests (IBM SPSS, Chicago, IL). All multivariate data analysis and modeling were accomplished using PCA and PLS-DA. PLS correlations were calculated to investigate the relationships between plasma lipid profile and the metabolomic profile of the hypercholesterolemic hamsters. Identification and quantification of metabolites was performed using Chenomx 7.6 software and the significance between the groups was determined using two-tailed Student *t*-test (Excel, Microsoft Office, Redmond, WA). Significance was defined at P < 0.05.

## Results

### Metabolic effects of high-fat diet and BaP supplementation

Animals were fed a high fat diet and water for the first four weeks of the study, the end point for the T0 group. After four weeks, groups T3, T7, T10, T14, and T21 received BaP as their drinking fluid for 3, 7, 10, 14, and 21 days, respectively, while continuing to consume the high fat diet. Animals were sacrificed at different time points, based on the number of days they were designed to receive BaP treatment. The net body weight gain for the first 4 weeks of the study, as well as the food efficiency ratio (g gained/g feed) was statistically significant in group T7 (P < 0.05, Table 2) when compared with controls. The ratio of fluid/water intake and the ratio of liver weight/body weight were not statistically different when compared with the T0 group (Table 2). While on treatment with BaP, all animals survived the duration of the study and no abnormal characteristics related to the physiology of the animals were noted.

**Table 2:** Anthropometrics of hypercholesterolemic male hamsters receiving BaP treatment for 0, 3, 7, 10, 14, and 21 days

	**T0**	**T3**	**T7**	**T10**	**T14**	**T21**
**Anthropometric data**						
Body weight gain, g/4 wks	32.7 ± 2.1	40.8 ± 3.7	50.6 ± 6.2^*^	44.2 ± 3.0	34.4 ± 3.5	39.8 ± 2.9
Food intake, g/d	7.1 ± 0.1	7.4 ± 0.2	7.5 ± .02	7.4 ± 0.2	6.9 ± 0.2	7.4 ± 0.1
Food efficiency ratio, g gain/g feed	0.16 ± 0.01	0.2 ± 0.02	0.22 ± 0.01^*^	0.2 ± 0.01	0.17 ± 0.01	0.19 ± 0.01
Fluid intake, PAZ/water	1.0 ± 0.0	0.96 ± 0.04	0.95 ± 0.05	1.1 ± 0.04	1.0 ± 0.06	1.1 ± 0.04
Liver *10^2^/body weight	4.6 ± 0.1	4.9 ± 0.1	4.7 ± 0.1	4.8 ± 0.1	4.7 ± 0.1	4.5 ± 0.2

Notes: Hamsters were fed high fat diet for 4 weeks, followed by high fat and BaP treatment for 0, 3, 7, 10, 14, and 21 days. Values are mean ± SE, N = 10 (T0) or N = 8 (T3, T7, T10, T14, and T21). * P < 0.05 when compared with T0 group. Statistical analysis ANOVA with Tukey’s procedure was used, SPSS software.

### Plasma lipid and lipoprotein profile

Plasma lipid profiles of the hypercholesterolemic hamsters were measured enzymatically to determine the therapeutic effect of BaP when administrated as the drinking fluid. Plasma TG and TC were not significantly reduced in the treatment groups, as compared with the control group (T0). However, the TC/HDL-C ratio was significantly lower in all treatment groups when compared with the control T0 group (P < 0.001, Table 3). The concentration of HDL-C was significantly increased in the T3 group (P < 0.05), as well as in the T21 group (P < 0.001), when compared with the controls. Consumption of BaP also significantly reduced the non-HDL cholesterol in all groups, as compared with the control animals (P < 0.01). These results corroborate with our previous findings that ingestion of PAZ and its biologically active fraction improves the plasma lipoprotein profile by significantly increasing the HDL-C concentrations, while decreasing the non-HDL cholesterol and TC/HDL-C ratio in hypercholesterolemic hamsters, both as preventative (previous study) and therapeutic (current study) agent.

**Table 3:** Plasma lipid concentrations in hypercholesterolemic male hamsters receiving BaP treatment for 0, 3, 7, 10, 14, and 21 days

	**T0**	**T3**	**T7**	**T10**	**T14**	**T21**
**Plasma lipids**						
Triglyceride, mmol/L	2.28 ± 0.15	2.34 ± 0.2	2.48 ± 0.38	2.72 ± 0.45	2.85 ± 0.41	2.51 ± 0.14
Total cholesterol, mmol/L	6.13 ± 0.18	5.22 ± 0.15	5.27 ± 0.12	5.32 ± 0.31	5.51 ± 0.22	6.07 ± 0.31
HDL cholesterol, mmol/L	2.69 ± 0.1	3.21 ± 0.17^*^	3.06 ± 0.12	3.03 ± 0.12	3.11 ± 0.01	3.7 ± 0.13^***^
Non HDL cholesterol, mmol/L	3.44 ± 0.1	1.94 ± 0.25^***^	2.18 ± 0.12^***^	2.26 ± 0.2^***^	2.39 ± 0.14^**^	2.37 ± 0.28^**^
TC/HDL cholesterol Ratio	2.3 ± 0.05	1.64 ± 0.1^***^	1.73 ± 0.06^***^	1.74 ± 0.05^***^	1.76 ± 0.03^***^	1.64 ± 0.08^***^

Notes: Hamsters were fed high fat diet for 4 weeks, followed by high fat and BaP treatment for 0, 3, 7, 10, 14, and 21 days. Values are mean ± SE, N = 10 (T0) or N = 8 (T3, T7, T10, T14, and T21). * P < 0.05; ** P < 0.01, *** P < 0.001 when compared with T0 group. Statistical analysis ANOVA with Tukey’s procedure was used, SPSS software.

### Hepatic mRNA gene expression of Apo A1

**Figure 1:**
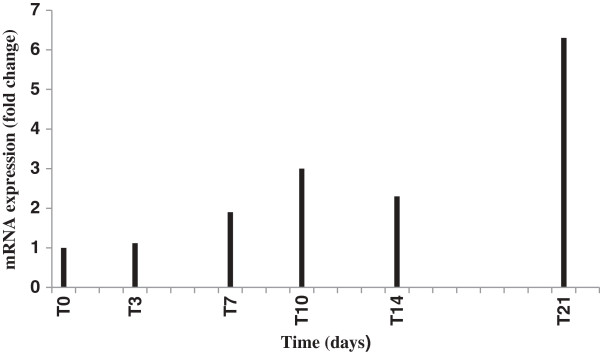
**Relative levels of hepatic mRNA expression of Apo A1 gene in hypercholesterolemic hamsters.** Animals were fed a high fat diet for 4 weeks, followed by high fat diet plus BaP for 0, 3, 7, 10, 14, and 21 days, respectively. Values were calculated as mean of threshold cycle values; N = 8 animals per group (N = 5 for T0 group). Each mRNA was normalized with GAPDH and is expressed as a fold change. Abbreviations: Apolipoprotein A1, ApoA1; Biologically active fraction of ProAlgaZyme, BaP; glyceraldehydes-3-phosphate dehydrogenase, GAPDH.

In order to determine the therapeutic potential of BaP at the molecular level and to validate the significant increase in HDL-C concentration, Apo A1 gene expression was evaluated. Also, the earliest time point at which the genetic expression is altered was determined. Apo A1 gene expression analysis showed that hamsters fed BaP for 10 days (T10) had a moderate (3-fold) increase, while hamsters fed BaP for 21 days exhibited an approximately 6-fold increase (Figure 1). This data correlates with the increase in the plasma concentration of HDL-C (Apo A1 being responsible for the production of nascent HDL particles). Thus the BaP increased HDL-C, at least in part, by increasing the production of nascent HDL particles, both when given as a therapeutic or preventative agent [7]. However, unlike the previous study where we saw a partial inhibition of CETP when PAZ was delivered as a preventative agent, no effects on CETP were apparent using the therapeutic approach within the timeframe of the current study (data not shown).

### Multivariate data analysis and metabolites identification on ^1^H NMR plasma

**Figure 2:**
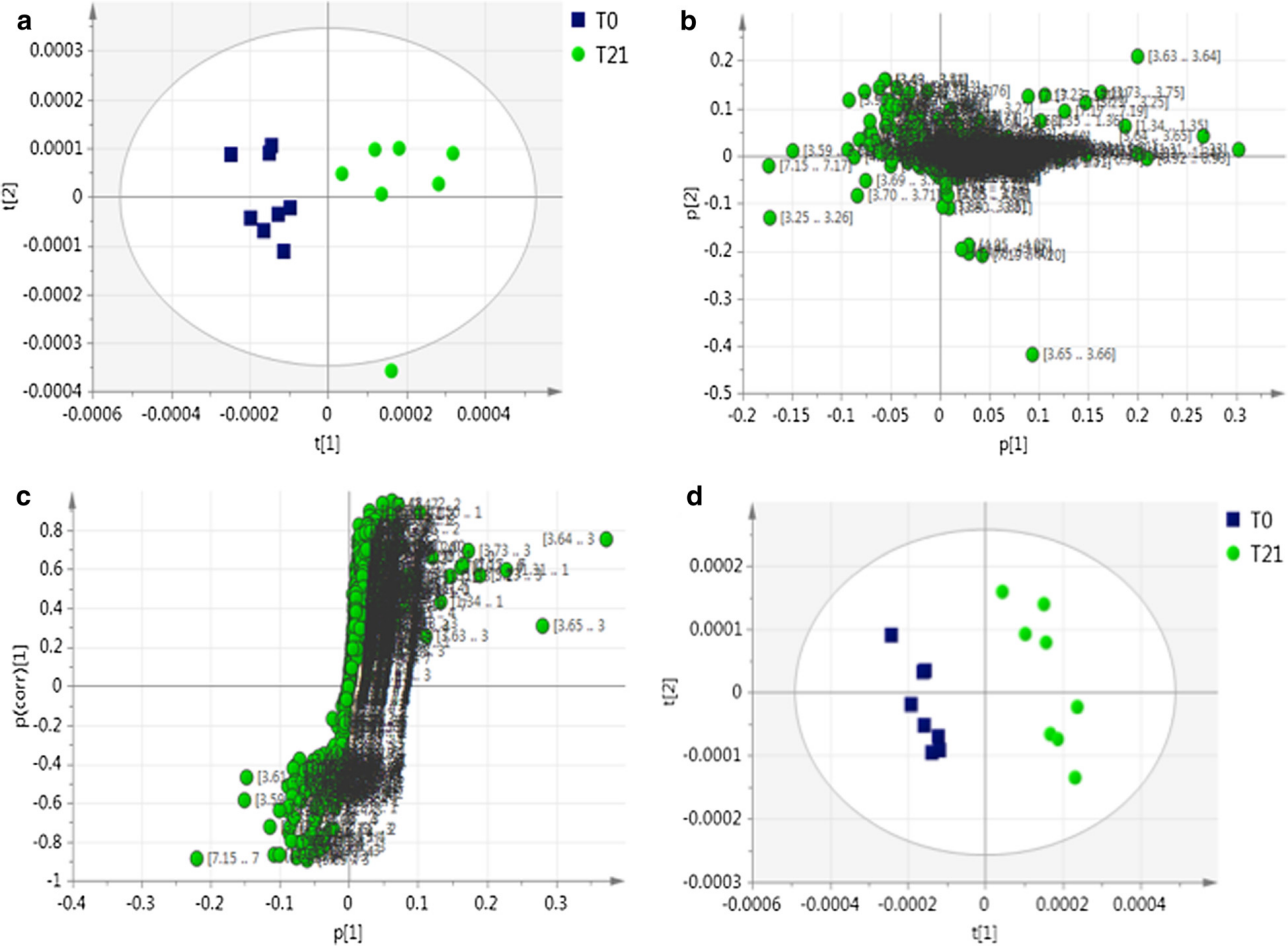
**Characterization of the plasma metabolomic changes induced by BaP.** 2**a.** CMPG_PCA score plot revealed that T0 and T21 groups were clearly separated along PC1 and PC2; 2**b.** CMPG_PCA corresponding loading plot indicating the regions of the spectra that are responsible for the group separation; 2**c.** OPLS_S-Plot used to identify possible metabolites that correlate the metabolomic profile to HDL-C concentration; 2**d.** Two-dimensional PLS-DA score plot showing group discrimination based on treatment received. Notes: T0, high fat diet and water; T21, high fat diet and BaP for 21 days. N = 8 hamsters per group. Abbreviations: Biologically active fraction of ProAlgaZyme, BaP; Carr-Purcell-Meiboom-Gill, CPMG; orthogonal projections to latent structures, OPSL; principal component, PC; principal components analysis, PCA; partial least squares-discriminant analysis, PLS-DA.

To further investigate the therapeutic effect of BaP on the hypercholesterolemic hamsters, plasma samples from controls (T0) and 21 days treatment group (T21) were subjected to metabolomic analysis using ^1^H NMR spectroscopy. PCA was performed on the NMR data to get overall information on metabolomic effects, if any, in hamsters due to BaP administration. The PCA score plots showed a clear separation in clusters produced by the T0 and T21 groups (Figure 2a), indicating differences in their plasma metabolomics profiles. Since one of the samples in the T21 group was outside the Hotelling’s 95% confidence interval (ellipse), it was considered to be a strong outlier and removed from the PCA analysis. The corresponding loading plot (Figure 2b) along with the S Plot (Figure 2c) provided important information on the contribution of each variable to the clustering pattern in the score plots. Both of these loading plots were used as a visual method to select and identify metabolites of interest for further analysis. In addition, the specific metabolites in the S-Plot reflected those strongly correlating with the changes in the plasma HDL-C concentrations.

**Figure 3:**
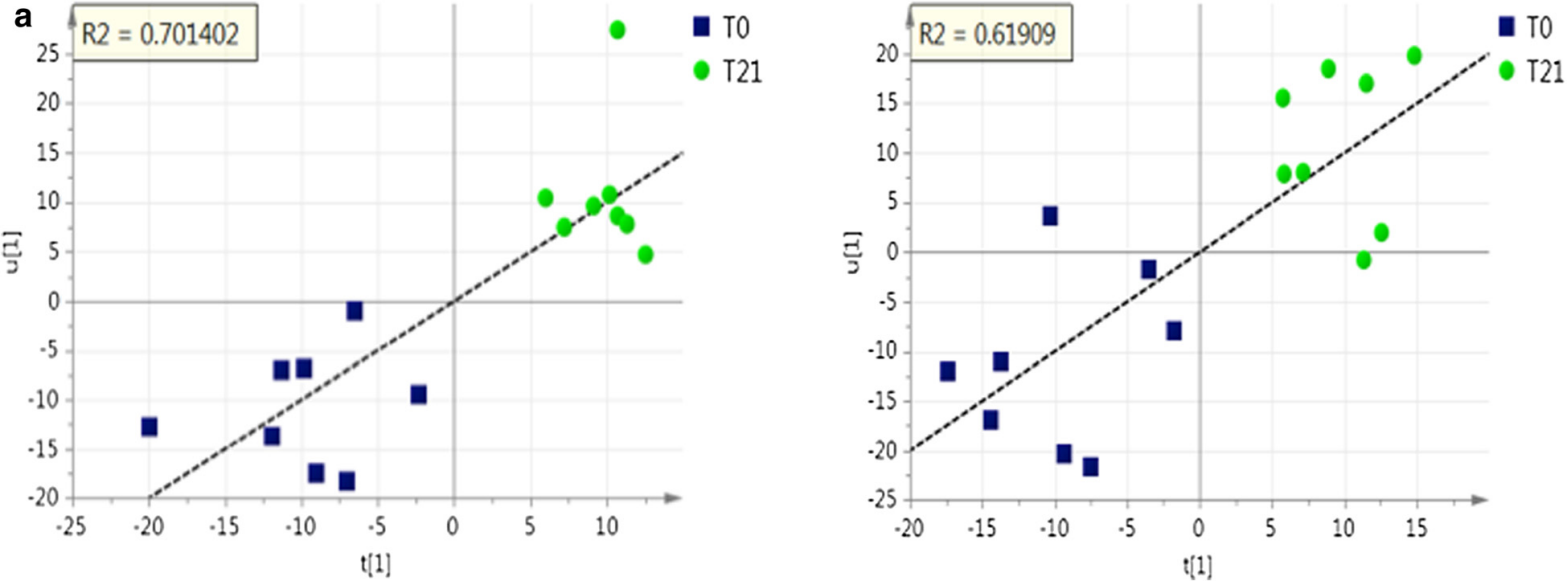
**Correlation of the BaP induced plasma metabolomic and lipid profile.** Changes among the metabolomic score values ([t1]) of individual hamsters fed a high fat diet and different drinking fluids (water or BaP) are plotted along with the corresponding individual plasma lipid profile scores ([u1]), as determined by the PLS analysis. The coefficient of determination (R^2^) among [t1] and [u1] that was calculated after linearization of the relationship is indicated. 3**a.** Relationship between the plasma metabolomic profile and complete lipid profile (R^2^ = 0.7) 3**b.** Relationship between the plasma metabolomic profile and plasma HDL-C (R^2^ = 0.62). Notes: T0, high fat diet and water; T21, high fat diet and BaP for 21 days. N = 8 hamsters per group. Abbreviations: Biologically active fraction of ProAlgaZyme, BaP; high density lipoprotein cholesterol, HDL-C; partial least squares, PLS.

The discriminant analysis (PLS-DA) indicated that the treatment with BaP for 21 days induced specific metabolomic patterns that enabled class assignment of the hamsters (PLS-DA, Figure 2d). The PLS-DA score plot revealed that the control and treatment group were clearly separated by principal component 1. In addition, the two-dimensional PLS plot (Figure 3a,b) allowed for the evaluation of BaP treatment on changes in the plasma metabolome. For instance, the treatment with BaP for 21 days induced a significant coefficient of determination R^2^ = 0.7, when the data were correlated with the plasma lipid profile (Figure 3a). The PLS regression between the NMR variables and the plasma lipid profile revealed that 70% of the variations in the lipid profile can be predicted by different treatment conditions (water vs. BaP). Furthermore, as determined by the PLS analysis, HDL-C concentrations were positively correlated with the plasma metabolomic profile (R^2^ = 0.62), indicating that the composition shift toward the higher density lipoproteins is reflected in the change in concentrations of some of the small metabolites present in plasma (Figure 3b).

A total of fifty plasma metabolites from the control and treatment groups were identified and quantified using Chenomx NMR Suite software, including amino acids (leucine, isoleucine, valine), organic acids (3-hydroxybutyrate, lactate, acetate, acetoacetate, citrate, pyruvate, creatine, creatinine), carbohydrates (glucose, galactitol, glucitol) and phospholipid-associated molecules. The metabolites that had a significantly lower concentration in the group that received BaP for 21 days are summarized in Table 4. Results indicated that the concentrations of choline, phosphocholine, glycerol-phosphocholine, betaine, and carnitine, were significantly lowered in T21 group as compared with T0. Treatment of hypercholesterolemic hamsters with BaP also resulted in decreased levels of several amino acids (arginine, leucine, isoleucine, threonine, taurine), as well as some other important molecules (3-hydroxy-butyrate, acetate, glycerol) involved in fatty acid metabolism.

**Table 4:** List of plasma metabolites quantified to be at lower concentrations after 21 days of treatment

**Metabolites**	**Peak regions (ppm)**	**Metabolites**	**Peak regions (ppm)**
2-Aminobutyrate	1.0,1.9,3.7	Glucitol	3.6, 3.7, 3.8
3-Hydroxybutyrate	1.2,2.3,2.4,4.1	Glycerol	3.6,3.8
Acetate	1.9	Isoleucine	0.9,1.0,1.2,1.5,2.0,3.7
Arabinitol	3.6,3.7,3.8,3.9	Lactate	1.3,4.1
Arginine	1.6,1.7,1.9,3.2,3.8	Leucine	0.9,1.7,3.7
Betaine	3.3,3.9	Phosphocholine	3.2,3.6,4.1
Carnitine	2.4,3.2,3.4,4.6	Glycero-phosphocholine	3.2,3.6,3.7,3.9,4.3
Choline	3.2,3.5,4.1	Taurine	3.2,3.4
Galactitol	3.7,4.0	Threonine	1.3,3.6,4.3

Notes: Plasma metabolites found to be significantly lower in T21 group as compared with T0 group. The metabolites were quantified using the Chenomx 7.6 NMR Suite database and significance was obtained using 2 tailed Student *t*-test, P < 0.05. T0, high fat diet and water; T21, high fat diet and BaP for 21 days, N = 4 hamsters/group for quantification of metabolites.

## Discussion

In our first study pertaining to PAZ, we determined that simultaneous administration of PAZ along with a high fat diet regimen improved the plasma lipid profile, by increasing the HDL-C concentrations and decreasing the non-HDL cholesterol, as well as the TC/HDL-C ratio. Also, the shift in lipoprotein concentration toward the higher density molecules was observed in the previous investigation. Moreover, the expression levels of important genes involved in HDL metabolism/reverse cholesterol transport were beneficially altered upon administration of PAZ and its biologically active subfraction [7].

The present study was designed to examine the *therapeutic* effect of BaP in the same diet-induced hypercholesterolemic hamster model used in our previous study [7]. More specifically, the objective of the current study was to determine the efficacy of BaP on plasma lipids and metabolomic profile of animals that had already attained a hypercholesterolemic state. Also, the aim was to document the earliest time point at which the plasma profile was modified after treatment with BaP. As this is the first study examining the plasma metabolomic profile of hypercholesterolemic hamsters treated with BaP, it is important to determine the changes in the concentration of small molecular weight metabolites following BaP therapy.

Due to interspecies differences in lipoprotein metabolism, it is essential to study metabolic changes in a non-primate model that develop the most similarities to the human disease. The hamster model used in our study has been previously found to be appropriate for exploring hypercholesterolemia associated with dietary changes [25,26] and the high fat diet used was previously reported to indeed induce hypercholesterolemia in hamsters [7]. Animals consumed similar amounts of diet and fluid throughout the study, however the hamsters in group T7 had a significant increase in body weight, as well as in the food efficiency ratio (g gain/g feed). Since we did not analyze body composition, we cannot speculate on the reason for this increase. The results of this therapeutic investigation reveal an improvement in the plasma lipoprotein profile upon administration of BaP, showing a significant reduction in non-HDL cholesterol and the TC/HDL-C ratio in all treatment groups, as well as a significant increase in plasma HDL-C concentration. In addition, there was a moderate (T7, T10, and T14 groups) and a highly significant increase (T21 group) in the hepatic mRNA levels of the Apo A1 gene, which is involved in the production of nascent HDL particles. Administration of PAZ and its biologically active fraction (BaP) showed a significant improvement in the plasma lipid profile, when fed to hypercholesterolemic hamsters. In our previous study, when PAZ or BaP were administered for 4 weeks as preventative agents, the hepatic mRNA expression for Apo A1, CETP, ATP-binding cassette transporter, member 1 (ABCA1), and scavenger receptor class B, member 1 (SRB1) were evaluated. However as the data only showed strong effects on Apo A1 and CETP (~ 5-fold increase in Apo A1 and 2-fold decrease in CETP expression), we focused only on these genes in the current study. In a trend similar to the earlier study, we saw an increase in the Apo A1 expression in this study as well. However, the CETP expression was not significantly inhibited in the timeframe of this study. Both studies also showed a lowering of non-HDL cholesterol. Whether or not BaP had any effect on cholesterol synthesis via the enzymatic activity of HMG-Co A reductase was not the focus of this study and hence not examined. Thus the mechanism by which non-HDL cholesterol may have decreased may be speculated, but needs further exploration.

Further, we analyzed the metabolomic plasma profile of hypercholesterolemic hamsters treated with BaP, using ^1^H NMR spectroscopy and multivariate data analysis. Previous studies have identified metabolomic perturbations associated with abnormal lipoprotein profile, kidney disease associated with type I diabetes, insulin resistance, and atherosclerosis using NMR spectroscopy - the latter has become a widely used method in the last decade [27,28].

Plasma includes both high molecular weight proteins and lipoproteins, as well as low molecular weight metabolites. Hence, the standard plasma one-dimensional ^1^H NMR spectrum is dominated by the broad resonance peaks from the high molecular weight components. The CPMG echo pulse technique was used to suppress the resonance from the macromolecules and emphasize the low molecular weight metabolites, thus revealing subtle biochemical information from the plasma samples. A clear separation of the groups was obtained with the PCA and PLS-DA score plots, showing changes in plasma metabolomic profiles due to BaP intervention. PCA loading plots were analyzed to identify the unique regions in the spectra and hence the metabolites responsible for the strong separation of the groups. In addition, the S-Plot generated a set of variables that are most reliably predictive of the changes in the plasma HDL-C concentration. Variables that are furthest away from the origin in the S-Plot are considered as potential biomarkers. The Chenomx NMR Suite software was used as the decisive tool in identification and qualification of important low molecular weight metabolites.

It is now well established that a high fat diet is highly correlated with an atherogenic outcome. Independent from the effect of a high fat diet on lipid profiles, a number of recent metabolomic studies have identified abnormalities in branched chain amino acids [29], choline, betaine, and trimethylamine N-oxide (TMAO) metabolism as being highly increased in subjects with atherosclerosis, heart failure, and other cardiovascular diseases [30-32]. After screening more than 2000 metabolites from a large cohort study (n = 1,876), Wang et al. [32] found that a unique cluster of three phospholipid-associated molecules, more specifically choline, betaine, and the final metabolite, TMAO are linked to or predictive of CVD risk. It has been shown that increased levels of these metabolites promoted up-regulation of several macrophage scavenger receptors that correlated with atherosclerosis, making these phospholipid metabolites independent predictors for the risk of a clinical vascular event. Even though cholesterol and triglycerides remain key culprits in atherosclerosis, the new findings have shifted the attention towards the phosphatidylcholine biosynthesis pathway for additional information related to risk for CVD. Plasma levels of choline and betaine are dependent on the proatherosclerotic phospholipid-rich diet, and are considered key risk factors, rather than direct markers of CVD [33]. After quantification of these metabolites in our plasma samples, we observed that the group which received the high fat diet and BaP for 21 days had significantly lower levels of betaine, choline, phosphocholine, and glycerol-phosphocholine, as compared with the animals that received the high fat diet and water.

Another recently studied metabolite that contains a trimethylamine structure similar to that of choline is L-carnitine. Its fundamental role is to transport fatty acids into the mitochondrial compartment [34], and it has been associated with potential health risk related to CVD [35]. It has been shown that TMAO, and its precursors choline and carnitine suppress in vivo reverse cholesterol transport, and elevated levels of plasma carnitine in humans are significantly associated with risk for coronary artery disease, peripheral artery disease, and overall CVD [36]. Our metabolomics data showed a significantly lower plasma concentration of carnitine in the group that received BaP for 21 days, as compared with the group that received a high fat diet and water. These findings, in conjunction with the increase in plasma HDL-C, along with the decrease in non-HDL cholesterol and the TC/HDL-C ratio, all considered contributory factors towards development of heart disease, are very exciting. It may be interesting to perform a follow up metabolomics analysis at the earlier time points of the study, to determine whether or not the changes in plasma lipids causes the changes in the metabolite concentrations or vice-versa.

## Conclusion

In this study we have demonstrated that the dietary intervention with BaP can cause a beneficial change in the lipid and metabolomic profile of diet induced hypercholesterolemia in hamsters. The metabolomic profile of the hamsters correlated strongly with the plasma lipid profile and HDL-C concentrations, thereby showing that BaP intake was beneficial in concomitant improvement of both the plasma lipid profile and the metabolomic profile. In addition to improving the established risk factors associated with CVD, mainly increasing HDL-C, and up-regulating the mRNA expression of the Apo A1 gene, we identified the potentially valuable effect of the algal infusion on relatively new predictors of atherosclerosis, namely betaine, carnitine, and choline. This research illustrates the power of the metabolomics approach to drive biomarker discovery and generate hypotheses for new treatments, thereby opening exciting avenues for future research.

## Abbreviations

Apo A1: Apolipoprotein A1; ABCA1: ATP-binding cassette transporter member 1; BaP: Biologically active fraction of ProAlgaZyme; CETP: Cholesteryl ester transfer protein; CPMG: Carr-Purcell-Meiboom-Gill; CVD: Cardiovascular disease; D2O: Deuterium oxide; EDTA: Ethylenediaminetetraacetic acid; GAPDH: Glyceraldehydes-3-phosphate dehydrogenase; HDL-C: High density lipoprotein cholesterol; LDL-C: Low density lipoprotein cholesterol; NMR: Nuclear magnetic resonance; OPLS: Orthogonal projections to latent structures; PAZ: ProAlgaZyme; PCA: Principal component analysis; PLS: Partial least squares; PLS-DA: Partial least squares-discriminant analysis; RT-PCR: Reverse transcriptase polymerase chain reaction; SRB1: Scavenger receptor class B member 1; TMAO: Trimethylamine N-oxide; TC: Total cholesterol; TG: Triglycerides.

## Competing interests

AG and SVG have rights to an application covering the biologically activity of PAZ. NS, PK and AGoja report no conflicts of interest with this work.

## Authors’ contributions

AG contributed to the study design, participated in sample collection and analysis, interpreted the data and drafted the manuscript. AGoja contributed to NMR data acquisition and multivariate data analysis. NS participated in sample collection and analysis. PK performed the cardiac puncture procedure and assisted with the preparation of the manuscript. SG, the PI, supervised all areas of the research, data analysis and manuscript. All authors read and approved the final manuscript.
